# Intermediate-to-therapeutic versus prophylactic anticoagulation for coagulopathy in hospitalized COVID-19 patients: a systemic review and meta-analysis

**DOI:** 10.1186/s12959-021-00343-1

**Published:** 2021-11-24

**Authors:** Sirui Zhang, Yupei Li, Guina Liu, Baihai Su

**Affiliations:** 1 West China School of Medicine, Sichuan University, 610041 Chengdu, China; 2 Department of Nephrology, Med+ Biomaterial Institute, West China Hospital, Sichuan University, 610041 Chengdu, China; 3 Institute for Disaster Management and Reconstruction, Sichuan University, 610207 Chengdu, China; 4 The first People’s Hospital of Shuangliu District, 610200 Chengdu, China; 5 Med-X Center for Materials, Sichuan University, 610041 Chengdu, China

**Keywords:** COVID-19, Anticoagulation, Mortality, Bleeding, Thromboprophylaxis, Meta-analysis

## Abstract

**Background:**

Anticoagulation in hospitalized COVID-19 patients has been associated with survival benefit; however, the optimal anticoagulant strategy has not yet been defined. The objective of this meta-analysis was to investigate the effect of intermediate-to-therapeutic versus prophylactic anticoagulation for thromboprophylaxis on the primary outcome of in-hospital mortality and other patient-centered secondary outcomes in COVID-19 patients.

**Methods:**

MEDLINE, EMBASE, and Cochrane databases were searched from inception to August 10th 2021. Cohort studies and randomized clinical trials that assessed the efficacy and safety of intermediate-to-therapeutic versus prophylactic anticoagulation in hospitalized COVID-19 patients were included. Baseline characteristics and relevant data of each study were extracted in a pre-designed standardized data-collection form. The primary outcome was all-cause in-hospital mortality and the secondary outcomes were incidence of thrombotic events and incidence of any bleeding and major bleeding. Pooled analysis with random effects models yielded relative risk with 95 % CIs.

**Results:**

This meta-analysis included 42 studies with 28,055 in-hospital COVID-19 patients totally. Our pooled analysis demonstrated that intermediate-to-therapeutic anticoagulation was not associated with lower in-hospital mortality (RR=1.12, 95 %CI 0.99-1.25, p=0.06, I^2^=77 %) and lower incidence of thrombotic events (RR=1.30, 95 %CI 0.79-2.15, p=0.30, I^2^=88 %), but increased the risk of any bleeding events (RR=2.16, 95 %CI 1.79-2.60, p<0.01, I^2^=31 %) and major bleeding events significantly (RR=2.10, 95 %CI 1.77-2.51, p<0.01, I^2^=11 %) versus prophylactic anticoagulation. Moreover, intermediate-to-therapeutic anticoagulation decreased the incidence of thrombotic events (RR=0.71, 95 %CI 0.56-0.89, p=0.003, I^2^=0 %) among critically ill COVID-19 patients admitted to intensive care units (ICU), with increased bleeding risk (RR=1.66, 95 %CI 1.37-2.00, p<0.01, I^2^=0 %) and unchanged in-hospital mortality (RR=0.94, 95 %CI 0.79-1.10, p=0.42, I^2^=30 %) in such patients. The Grading of Recommendation, Assessment, Development, and Evaluation certainty of evidence ranged from very low to moderate.

**Conclusions:**

We recommend the use of prophylactic anticoagulation against intermediate-to-therapeutic anticoagulation among unselected hospitalized COVID-19 patients considering insignificant survival benefits but higher risk of bleeding in the escalated thromboprophylaxis strategy. For critically ill COVID-19 patients, the benefits of intermediate-to-therapeutic anticoagulation in reducing thrombotic events should be weighed cautiously because of its association with higher risk of bleeding.

**Trial registration:**

The protocol was registered at PROSPERO on August 17th 2021 (CRD42021273780).

**Graphical abstract:**

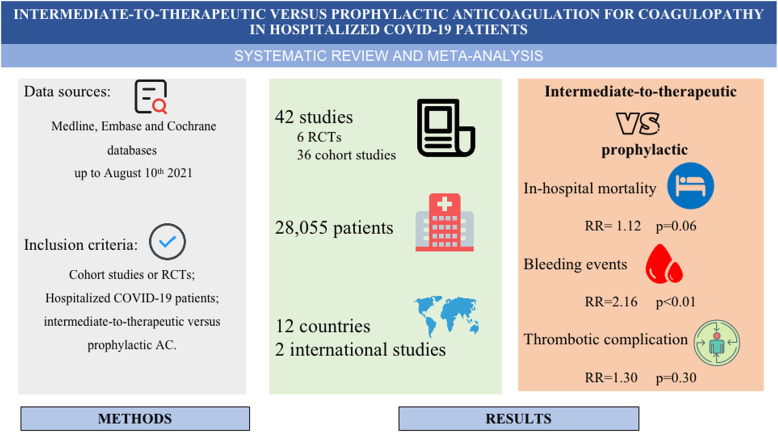

**Supplementary Information:**

The online version contains supplementary material available at 10.1186/s12959-021-00343-1.

## Background

Coronavirus disease 2019 (COVID-19), provoked by severe acute respiratory syndrome coronavirus-2 infection, is currently the most serious public health crisis worldwide [1]. Patients who are hospitalized with COVID-19 frequently have macrovascular and microvascular thrombosis and inflammation, which may contribute to high morbidity and mortality [2–7]. In a recent meta-analysis of 49 studies enrolling 18,093 hospitalized patients with COVID-19, the overall estimated pooled incidence of venous thromboembolism was 17.0 %, with a higher incidence (27.9 %) among those admitted to intensive care unit (ICU) [8]. Mechanisms of hypercoagulable state in these patients have not been fully elucidated yet, and may potentially attribute to immunothrombosis that is mediated by abnormal endothelial dysfunction and platelet activation [9–11]. Accordingly, several national and international clinical guidance reports have recommended the use of prophylactic anticoagulation in hospitalized COVID-19 patients who do not have a contraindication to anticoagulant administration for thromboprophylaxis [12–15].

However, increasing evidence demonstrated that some COVID-19 patients, especially those with critical illness, still developed severe thrombotic complications despite the use of prophylactic anticoagulant [16–18]. Enhanced-dose anticoagulation strategies have thus been recommended by some guidance statements in critically ill COVID-19 patients [19–21], though patients may also develop bleeding complications on elevated anticoagulation treatment. The effectiveness and safety of intermediate-to-therapeutic anticoagulation are still uncertain [22].

Previous meta-analyses that investigated the optimal anticoagulation strategy only included limited studies and reported inconsistent conclusions [23–25]. As more evidence from high-quality randomized controlled trials (RCTs) and observational cohort studies have become available recently, we conducted an updated meta-analysis to investigate the efficacy and safety of intermediate-to-therapeutic versus prophylactic anticoagulation in COVID-19 patients with subsequent subgroup analyzes being performed in critically ill COVID-19 patients further.

## Methods

This review was conducted following the Preferred Reporting Items for Systemic Reviews and Meta-Analyses (PRISMA) Statement [26] and registered on the Open PROSPERO Framework (registration number: CRD42021273780).

### Search strategy

Literature search was conducted strictly and comprehensively in MEDLINE, EMBASE, and Cochrane databases. Two independent investigators (ZSR and LYP) searched studies available from database inception to August 10th 2021 without language limitation. The following key words and/or medical subject heading terms were used: ‘COVID’, ‘COVID-19’, ‘2019 novel coronavirus infection’, ‘SARS-CoV-2’, ‘2019-nCoV disease’, ‘anticoagulation’, ‘anticoagulants’, ‘anticoagulant’, ‘unfractionated heparin’, ‘UFH’, ‘fondaparinux’, ‘enoxaparin’, ‘low-molecular-weight heparin’, ‘LMWH’, ‘heparin, low molecular weight’, ‘heparin’, ‘antithrombotic’, and ‘anti-thrombosis’, ‘thromboprophylaxis’ [see Additional file 1 for the detailed search strategy].

### Study selection

Two independent investigators (ZSR and LYP) performed the initial screening of titles and abstracts. Full-length articles of identified studies were retrieved to assess eligibility. Any discrepancies were adjudicated by a third reviewer (SBH). The inclusion criteria of our meta-analysis were as follows: (1) cohort studies and RCTs, (2) studies that enrolled COVID-19 patients admitted to general wards or ICUs, and (3) studies that compared the efficacy or safety of intermediate-to-therapeutic versus prophylactic anticoagulation. The exclusion criteria were as follows: (1) studies enrolling out-hospital COVID-19 patients, or (2) animal experiments, case reports, reviews, comments, editorial comments, or (3) no relevant and sufficient data on preferred outcomes. In case a same patient population was enrolled in a few articles, we only selected the most comprehensive study.

### Data extraction

After study selection, the following data were extracted: author, publication year, study type, study location, patient characteristics, numbers of patients in different groups, in-hospital mortality, incidence of any bleeding events, incidence of major bleeding events, and incidence of thrombotic events using a predesigned standardized data-collection form by two researchers (ZSR and LYP) independently.

### Quality assessment

Quality assessment of studies were conducted by two independent reviewers (ZSR and LYP). The Cochrane Collaboration tool was used to assess the risk of bias in randomized controlled trials [27]. The quality of cohort studies was assessed using the Newcastle–Ottawa Scale (NOS) [28], evaluating three aspects: (1) selection of study groups, (2) comparability of study groups, and (3) outcome ascertainment. Meanwhile, we rated the quality of evidence for each outcome in the pooled analysis as high, moderate, low, and very low quality by the Grading of Recommendations Assessment, Development and Evaluation (GRADE) framework [29].

### Study outcomes

Our primary outcome was all-cause mortality during hospitalization for COVID-19. Secondary patient-centered outcomes included the incidence of bleeding events and thrombotic complications during hospitalization for COVID-19. We also investigated these outcomes in critically ill COVID-19 patients who were admitted to ICU exclusively. Definitions of bleeding events depended on the variable definitions of each study, mostly in accordance with Myocardial Infarction (TIMI) bleeding [30] or the World health organization bleeding scale [31, 32]. Major bleeding was mostly defined by the criteria of the International Society on Thrombosis and Haemostasis (ISTH) [33]. Thrombotic complications mainly included deep venous thrombosis and pulmonary embolism, or jointly venous thromboembolism.

### Statistical analysis

Risk ratios (RR) with 95 % confidence interval (CI) were calculated for dichotomous outcomes such as mortality, bleeding events and thrombotic complications. A random effects model was applied for meta-analyses due to the heterogeneity of study populations and design. Heterogeneity among studies was assessed by the chi-square test and shown as an I^2^ index (25–50 %: low heterogeneity; 50–75 %: moderate heterogeneity; greater than 75 %: high heterogeneity). Sensitivity analysis was conducted by omitting each study at a time to determine whether each study affected the overall estimate and to identify studies that potentially drove the results. Publication bias was assessed with funnel plots and Egger test. A two-side P-value <0.05 was considered to be of statistical significance. All statistical analyses were carried out using the meta package in R 3.6.2 and Review Manager software (version 5.4).

## Results

### Study Selection

**Fig. 1:**
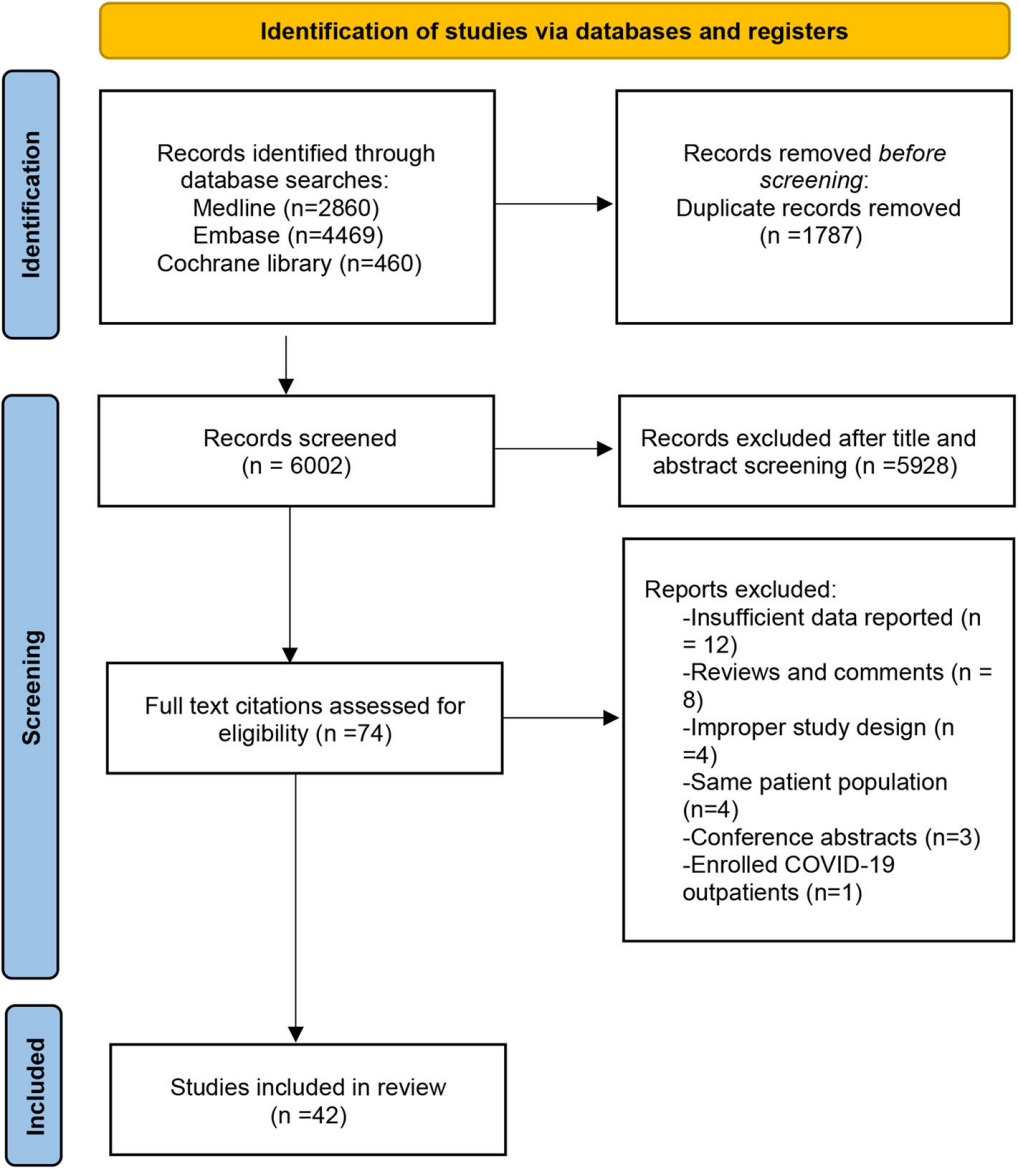
Flowchart of selection of studies

From 6,002 yielded studies, we retained 74 studies after removing duplications and screening titles and abstracts. Forty-two studies involving 28,055 subjects met the inclusion criteria and were finally included in this meta-analysis after full-text review. The PRISMA flow diagram demonstrates the process of study screening and selection in detail, as shown in Fig. 1.

## Study Characteristics

Of the forty-two included studies, 6 studies [34–39] were RCTs and 36 studies [40–75] were cohort studies. Thirteen studies [34, 38, 39, 41, 43, 52, 55, 58, 61, 64, 67, 69, 70] were performed exclusively in patients admitted to ICU. Eighteen studies [34, 40, 42, 48, 51, 53–55, 57, 62–66, 68–70, 73] reported results from the United States and two [37, 38] were international studies. Other studies originated from eleven countries ( Italy [47, 49, 56, 58, 60, 72, 75], France [41, 61, 67], Brazil [35, 36], England [59], Greek [46], China [45], Japan [43], Mexico [44], United Arab Emirates [52, 71], Turkey [74], Iran [39] and Spain [50] ).

We included a pooled population of 23,579 hospitalized COVID-19 patients [34–49, 51–61, 63–68, 70–75] with reported information related to in-hospital mortality, 19,275 patients [34–41, 44, 46–48, 50, 51, 53, 55, 58, 59, 61, 62, 64–67, 70, 71, 73] with reported information related to bleeding events and 8492 patients [34–39, 41–43, 47, 56, 58, 60–62, 64, 66] with reported information related to thrombotic events. The mean age in most studies was over 60 years. The proportion of male subjects ranged from 38.1 to 82.2 %. The types of administered anticoagulants, exact anticoagulation dosages and anticoagulation treatment duration varied widely across studies. Enoxaparin and unfractionated heparin were the most used anticoagulants in the included studies. Additional file 2 further summarized the detailed baseline characteristics of each study and the details of anticoagulation administration in each study were listed in Additional file 3.

## Assessment of study quality

The score by NOS for the included cohort studies ranged from 6 to 9, while 10 studies were of high quality scoring 9. A full assessment is shown in Additional file 4. Cochrane Collaboration Risk of Bias assessing bias of RCTs indicated low bias in the majority of included RCTs (Additional file 5).

## In-hospital mortality

**Fig. 2:**
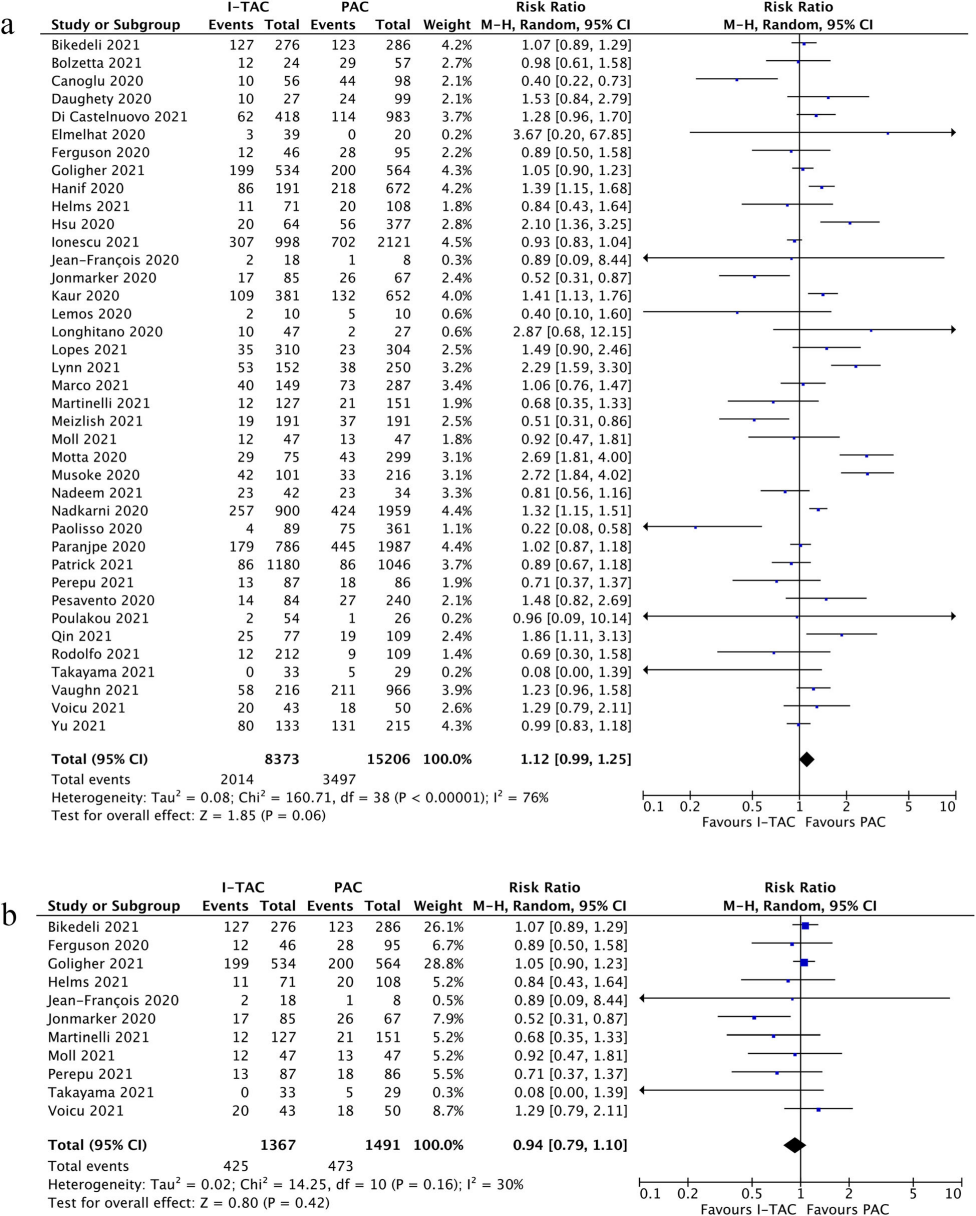
**a** Forest plot of the effect of intermediate-to-therapeutic versus prophylactic anticoagulation on in-hospital mortality in general COVID -19 patients; **b** Forest plot of the effect of intermediate-to-therapeutic versus prophylactic anticoagulation on in-hospital mortality in COVID -19 patients admitted to ICU

Data regarding in-hospital mortality of COVID-19 patients receiving prophylactic anticoagulation or intermediate-to-therapeutic anticoagulation were available from 39 studies [34–49, 51–61, 63–68, 70–75] (23,579 patients) which included both ICU and non-ICU patients. In-hospital mortality ranged widely across studies with an average of 22.1 % for prophylactic anticoagulation group and 22.6 % for intermediate-to-therapeutic anticoagulation group in non-ICU patients, while the average incidence of in-hospital death was 31.7 % in prophylactic anticoagulation group and 31.1 % in intermediate-to-therapeutic anticoagulation group in ICU patients. Figure 2a demonstrated that intermediate-to-therapeutic anticoagulation was not significantly associated with reduced in-hospital mortality compared to prophylactic anticoagulation in unselected hospitalized COVID-19 patients (RR=1.12, 95 %CI 0.99-1.25, p=0.06, I^2^=77 %). The sensitivity analysis by leave-one-out approach indicated that the result was relatively stable because the risk ratio remained unchangeable though p value fluctuated around 0.05, as shown in Additional file 6. The funnel plot and Egger test (p=0.79) showed no evidence of significant publication bias, as shown in Additional file 7. The quality of evidence for in-hospital mortality for general COVID patients was rated as very low using GRADE framework in view of high heterogeneity and potential selection bias (Additional file 8).

Eleven studies [34, 38, 39, 41, 43, 55, 58, 61, 64, 67, 70] exclusively investigated in-hospital mortality of critically ill COVID-19 patients admitted to ICU. The subgroup analysis of ICU patients also indicated that intermediate-to-therapeutic anticoagulation was not significantly associated with reduced in-hospital mortality when compared with prophylactic anticoagulation and heterogeneity across the studies decreased significantly (RR=0.94, 95 %CI 0.79-1.10, p=0.42, I^2^=30 %, see Fig. 2b). The quality of evidence for subgroup analysis of ICU settings regarding in-hospital mortality were rated as moderate-quality, as shown in Additional file 8. In the subgroup analysis stratified by study regions, we also did not observe a significant survival benefit in patients receiving intermediate-to-therapeutic anticoagulation as compared with those receiving prophylactic anticoagulation although the heterogeneity across different subgroups was high (see Additional file 9).

## Bleeding events

**Fig. 3:**
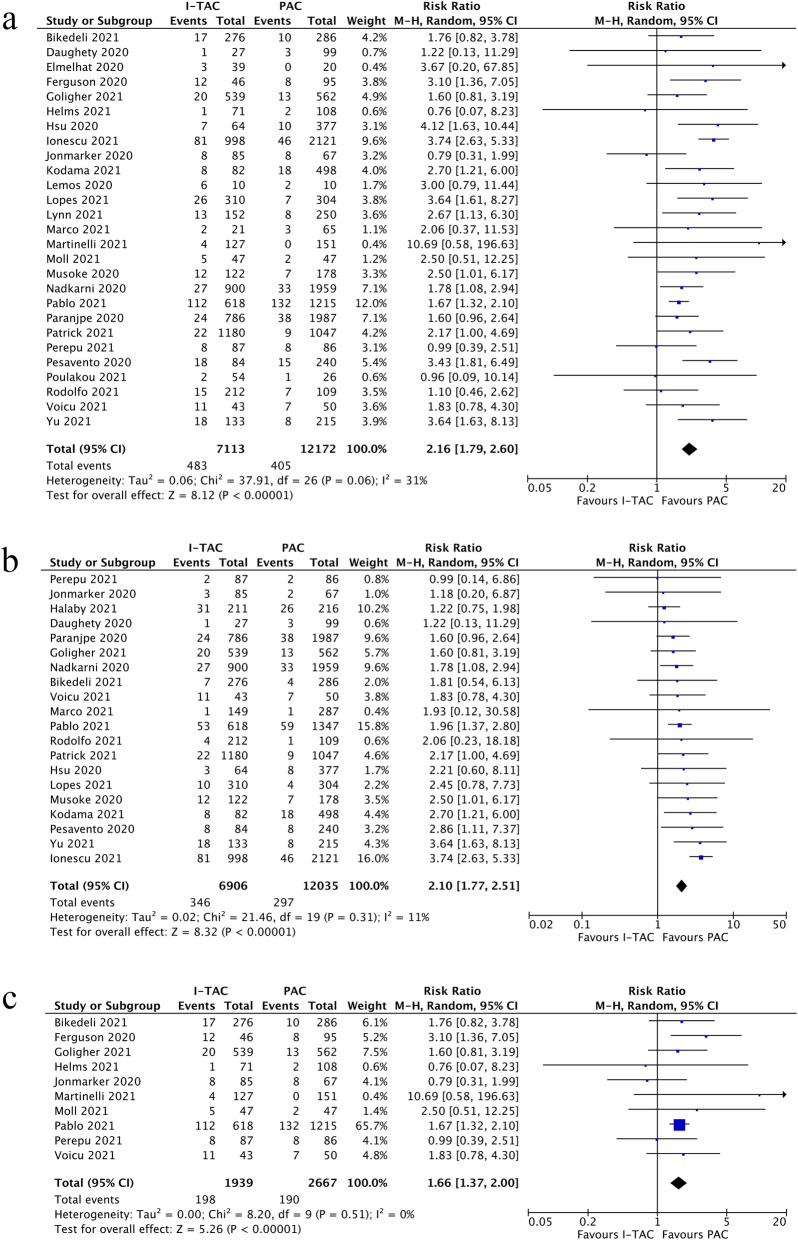
**a** Forest plot of the effect of intermediate-to-therapeutic versus prophylactic anticoagulation on any bleeding events in general COVID -19 patients; **b** Forest plot of the effect of intermediate-to-therapeutic versus prophylactic anticoagulation on major bleeding events in general COVID-19 patients; **c** Forest plot of the effect of intermediate-to-therapeutic versus prophylactic anticoagulation on any bleeding events in COVID-19 patients admitted to ICU

A total of 27 studies [34–41, 44, 46–48, 50, 51, 53, 55, 58, 59, 61, 62, 64–67, 70, 71, 73] including 19,275 patients assessed the incidence of any bleeding events in intermediate-to-therapeutic anticoagulation group and prophylactic anticoagulation group. COVID-19 patients admitted to general wards, the average incidence of any bleeding events was 2.27 % in prophylactic anticoagulation and 5.50 % in intermediate-to-therapeutic anticoagulation group. In ICU settings, the average bleeding incidence was 7.14 % in prophylactic anticoagulation group and 10.2 % in intermediate-to-therapeutic anticoagulation group. As shown in Fig. 3a, intermediate-to-therapeutic anticoagulation was significantly associated an increased incidence of any bleeding events when compared to prophylactic anticoagulation (RR=2.16, 95 %CI 1.79-2.60, p<0.01, I^2^=31 %). The predefined sensitivity analyses did not change the overall effect and the results remained stable (Additional file 10). Besides, evidence of publication bias was not found according to the funnel plot and following Egger test (p=0.757) (Additional file 11). The quality of evidence for bleeding events was rated as moderate using GRADE framework, as shown in Additional file 12. Figure 3b further showed that intermediate-to-therapeutic anticoagulation was also associated with higher incidence of major bleeding events significantly (RR=2.11, 95 %CI 1.77-2.51, p<0.01, I^2^=11 %).

Subgroup analysis of bleeding risk related to thromboprophylaxis in ICU settings included 10 studies [34, 38, 39, 41, 50, 55, 58, 61, 64, 70] and 4596 patients totally, showing that intermediate-to-therapeutic anticoagulation was significantly associated with increased incidence of any bleeding events with low heterogeneity (RR=1.66, 95 %CI 1.37-2.00, p<0.01, I^2^=0 %, Fig. 3c). The quality of evidence for subgroup analyses of ICU settings regarding bleeding incidence were rated as moderate-quality evidence respectively, as shown in Additional file 12.

## Thrombotic complication events

**Fig. 4:**
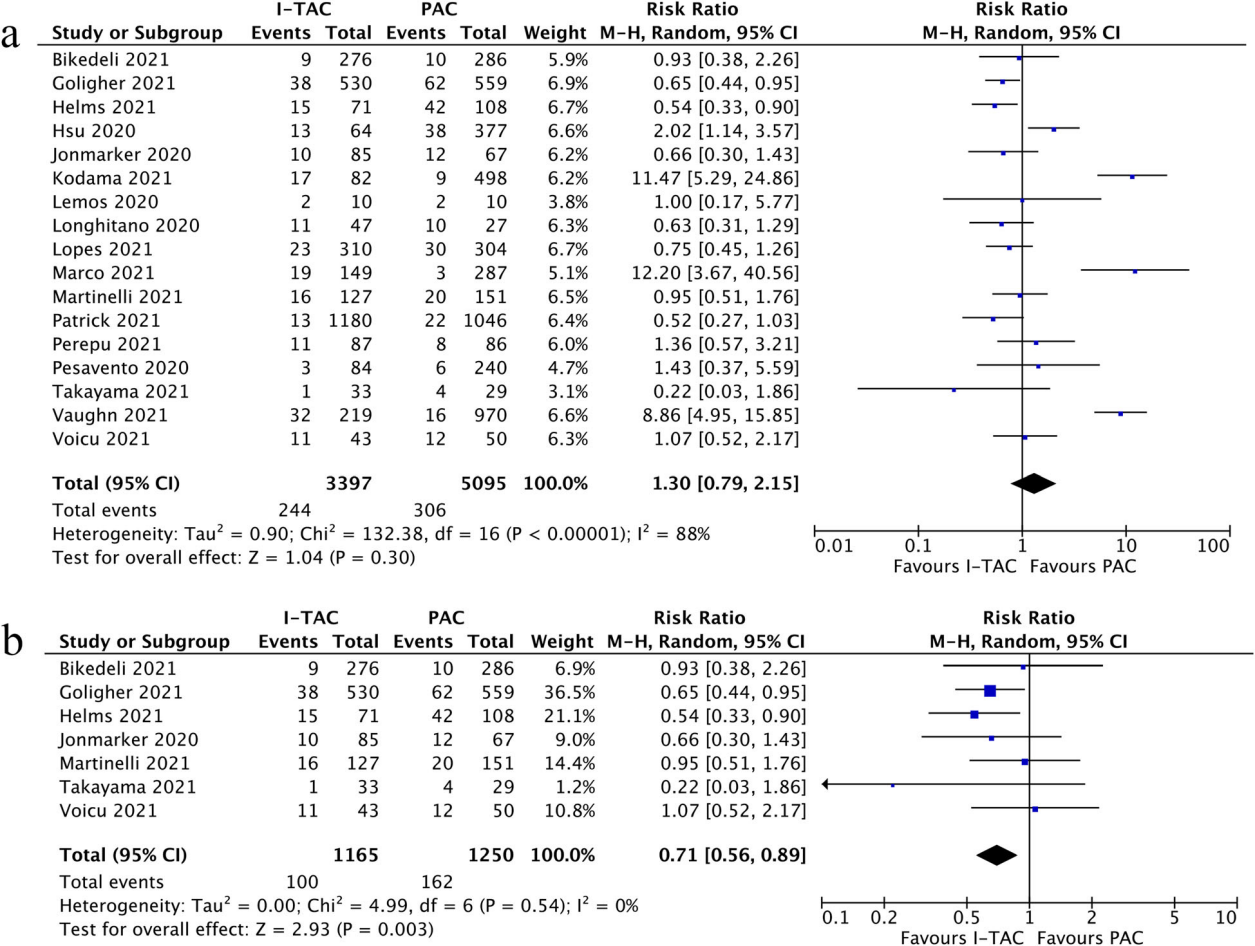
**a** Forest plot of the effect of intermediate-to-therapeutic versus prophylactic anticoagulation on thrombotic events in general COVID-19 patients; **b** Forest plot of the effect of intermediate-to-therapeutic versus prophylactic anticoagulation on thrombotic events in COVID-19 patients admitted to ICU

Incidence of thrombotic events was reported in 17 studies [34–39, 41–43, 47, 56, 58, 60–62, 64, 66] which included a total of 8,492 admitted patients with COVID-19. The results ranged widely across studies with an average incidence of 3.75 % for prophylactic anticoagulation group and 6.45 % for intermediate-to-therapeutic anticoagulation group in non-critically ill patients, while 13.0 % for prophylactic group and 8.58 % for intermediate-to-therapeutic anticoagulation group in ICU patients. As shown in Fig. 4a, the current meta-analysis found that the pooled risk ratio of thrombotic events risk did not favor either of two groups with high heterogeneity (RR=1.30, 95 %CI 0.79-2.15, p=0.30, I^2^=88 %). The predefined sensitivity analysis conformed the stability of the results in this meta-analysis, as shown in Additional file 13. The funnel plot and following Egger test (p=0.45) further indicated no evidence of significant publication bias (Additional file 14). The quality of evidence for thrombotic events was rated as very low using GRADE framework, as shown in Additional file 15.

Subgroup analysis of studies exclusively enrolling critically ill COVID-19 patients admitted to ICU indicated that patients receiving intermediate-to-therapeutic anticoagulation were associated with reduced risk of thrombotic events compared with prophylactic anticoagulation (RR=0.71, 95 %CI 0.56-0.89, p =0.03, I^2^=0 %), as shown in Fig. 4b. The quality of evidence for these subgroup analyses were rated as moderate-quality for ICU settings, as shown in Additional file 15.

## Discussion

In this systemic review and meta-analysis, we included 42 studies with 28,055 COVID-19 patients admitted to hospital in total. By analyzing data of 23,579 hospitalized COVID -19 patients from 33 cohort studies and 6 RCTs reporting in-hospital mortality, intermediate-to-therapeutic anticoagulation did not improve the primary outcome of in-hospital mortality in unselected hospitalized COVID-19 patients when compared with prophylactic anticoagulation. Additionally, a subgroup analysis of critically ill COVID-19 patients admitted to ICU also showed no significant survival benefit of intermediate-to-therapeutic anticoagulation against standard thromboprophylaxis. These results were inconsistent with earlier studies which reported a significant association between enhanced-dose anticoagulation and improved outcomes [51, 65]. It is noteworthy that the average in-hospital mortality for non-critically ill COVID-19 patients was 22.1 % for prophylactic anticoagulation group and 22.6 % for intermediate-to-therapeutic anticoagulation group in our meta-analysis, suggesting a relatively moderate-to-severe illness in such patients where the underlying thrombotic states and inflammatory damage may have been too advanced to be influenced and improved by higher doses of anticoagulants. In contrast, the latest adaptive, multiplatform, controlled trial (ATTACC, ACTIV-4a and REMAP-CAP) found that therapeutic-dose anticoagulation was associated with lower mortality until hospital discharge with a reduced need for organ support in the moderately ill COVID-19 patients with a crude 28-day in-hospital mortality of only 8.2 % for prophylactic anticoagulation group and 7.3 % for intermediate-to-therapeutic anticoagulation group [37]. Beyond clinical settings, different study countries might also have different discernible outcomes. As shown in Additional file 9, our subgroup analysis classified by study regions found that intermediate-to-therapeutic anticoagulation was not associated with lower in-hospital mortality in the pooled analysis of studies from South America, Europe, East Asia and West Asia. Data from North America even showed a significant survival benefit of prophylactic anticoagulation in unselected hospitalized COVID-19 patients as compared with intermediate-to-therapeutic anticoagulation. In general, the available evidence showed that therapeutic anticoagulation did not reduce in-hospital mortality in both critically ill and non-selected patients with COVID-19, and the evidence was of moderate quality for severe COVID-19 patients admitted to ICU and of very low quality for general COVID-19 patients admitted to hospital. Heterogeneity across the included studies was high, which might derive from different study design, imbalances in baseline characteristics, varied anticoagulation therapy (e.g., drug type, exact dosage, and route), diverse disease severity or different study countries.

Additionally, meta-analysis of data from 11 cohort studies and 6 RCTs reporting thrombotic events indicated that intermediate-to-therapeutic anticoagulation did not reduce relative risk of thrombotic events either versus prophylactic anticoagulation in general COVID-19 patients. However, a subgroup analysis of critically ill patients admitted to ICU found intermediate-to-therapeutic anticoagulation significantly reduced the incidence of thrombotic events by 30 %. These results should be interpreted with caution considering that possible insufficient thrombosis screening in patients admitted to general wards might inevitably influence the reliability of this evidence [76].

Recent studies have also reported high incidences of hemorrhage among inpatients with COVID-19. In this meta-analysis, we observed a significant increase of incidence of any bleeding events and major bleeding events in both general and critically ill patients receiving intermediate-to-therapeutic anticoagulation when compared with prophylactic anticoagulation. Especially, in critically ill COVID-19 patients, use of intermediate-to-therapeutic dose anticoagulants significantly reduced incidence of thrombotic events by 30 % but increased incidence of bleeding events by nearly 70 %, without a beneficial effect on patient survival. Therefore, the potential profit of elevated dose anticoagulation of thromboprophylaxis on thrombosis prevention are associated with an increase in bleeding events and should thus be weighed against the risk of bleeding.

Growing evidences have arisen to determine the optimal anticoagulation strategy that weighs thrombotic events and consequent bleeding risk in hospitalized patients with COVID-19. In the updated guidelines on the use of anticoagulants for thromboprophylaxis in COVID-19 patients, the American Society of Hematology recommended prophylactic anticoagulation over intermediate anticoagulation in COVID-19 patients with no confirmed or suspected thromboembolism based on low certainty of the evidence [77]. The latest NICE guideline also recommended a standard prophylactic dose of a low molecular weight heparin to young people and adults with COVID-19 who need low-flow or high-flow oxygen, continuous positive airway pressure, non-invasive ventilation or invasive mechanical ventilation within 14 h of admission [78]. Consistently, our meta-analysis further confirmed the insufficient beneficial effects of high-dose anticoagulation in general COVID-19 patients admitted to hospital. It is also noteworthy that the main outcomes in the included RCTs were typically composite endpoints [35, 37, 38, 79], which were not necessarily all related to the same end goal and limited the potential impact of these results in our daily clinical practice [76]. We instead focused on single endpoints like in-hospital mortality, thrombotic events and bleeding events. Therefore, the fact that therapeutic anticoagulation improved the composite outcome involving survival and receipt of organ support among non-critically ill patients in the multiplatform trials [37] is not as such recognized in the present review.

COVID-19 patients may exhibit complex coagulopathy states. Thrombotic and bleeding events are staggered in time and hypercoagulable states are not always consistent in the course of disease. A recent literature review by Tacquard, et al. demonstrated that thrombotic events occurred primarily in the first ten days after admission while bleeding events occurred most often late [76]. Hardy et al. also found an increase in thrombin generation with a decrease in overall fibrinolytic capacity during the first week of hospitalization, resulting in a strong procoagulant state. After this early stage of the disease, inflammatory markers and D-dimer levels gradually decreased in survivors, probably in relation to a decrease in the intensity of processes leading to microthrombosis [80]. Thromboprophylaxis strategy in hospitalized COVID-19 patients should thus be related to the disease progression and vary according to the severity of illness. D-dimers and fibrin monomers, another fibrin-related biomarker, have been extensively studied in COVID-19, and elevated levels of such biomarkers are associated with increased disease severity and mortality [81–83]. Recently, Godon, et al. showed that D-dimers and fibrin monomers were both useful to predict thrombotic events in COVID-19 patients [82]. The optimal cutoff value was determined at 5700 µg/L for fibrin monomers to predict thrombotic events with a sensitivity of 67 % and a specificity of 77 %, while the optimal cutoff value for D-dimers was 3300 µg/L with a sensitivity of 75 % and a specificity of 71 % [82]. Besides, these biomarkers are also of great value to adapt thromboprophylaxis protocol for coagulopathy in COVID-19 patients. Tassiopoulos et al. found D-dimer-driven anticoagulation protocol significantly reduced the overall mortality (31 % vs. 57 %) in intubated COVID-19 patients using a propensity-matched analysis [84]. Julie et al. further established an individualized, targeted‑intensity pharmacologic thromboprophylaxis protocol evaluating degree of illness severity, total body weight, and biomarkers (involving D-dimers and thromboelastography max amplitude) in 803 COVID‑19 patients admitted to hospital, and found that patients in the targeted-intensity thromboprophylaxis protocol group experienced significantly fewer thrombotic events, fewer major bleeding events, and lower mortality versus patients treated by standard thromboprophylaxis protocol [85]. These results encouraged the clinicians to adopt an individualized and targeted approach to escalated anticoagulation regimens for coagulopathy in COVID-19 patients admitted to both ICU and ward settings in future studies.

Importantly, the choices of different anticoagulation doses mainly follow local institutional protocols and were decided by the physicians in most studies, which resulted in the inconsistent anticoagulation dosage within the same group leading to potential bias [86]. For instance,in the ATTACC, ACTIV-4a and REMAP-CAP trial including critically ill COVID-19 patients, 22.4 % patients among therapeutic-dose group did not receive therapeutic-dose anticoagulation, whereas 51.7 % patients among control group received an intermediate dose, which may blunt the potential benefit of therapeutic-dose anticoagulation [38, 86]. Another challenge of precise anticoagulation in some COVID-19 patients is heparin resistance that occurs with unfractionated heparin only and entails the need of huge unfractionated heparin dosages to reach the therapeutic target [87, 88]. Therefore, monitoring of anticoagulation with anti-Xa activity is warranted to prevent a possible increased bleeding risk when high dosages of heparin are administered [87, 89].

This study has several limitations. Firstly, we included 36 cohort studies and only 6 RCTs. In those cohort studies, selection bias exists. Patients with higher disease severity and risk of thrombotic events were more likely to be treated by intermediate-to-therapeutic anticoagulation, which might lead to undervaluation of potential benefit. Secondly, most patients enrolled in the included studies were over 60 years old and it will be more reasonable to include patients with a wider age scope considering mortality is significantly higher in the elderly inpatients with COVID-19. Thirdly, the criteria for admission to hospital, definition of in-hospital mortality, bleeding and thrombotic events, follow-up periods and exact dosage of two groups might vary from region to region during different stages of the pandemic, affecting risk of both thrombotic and fatal outcomes. Results of the current meta-analysis should therefore be interpreted with caution considering these limitations.

## Conclusions

Among unselected in-hospital patients with COVID-19, the current meta-analysis showed that intermediate-to-therapeutic anticoagulation was not associated with lower in-hospital mortality and incidence of thrombotic events, but increased the risk of bleeding events versus prophylactic anticoagulation. In contrast, intermediate-to-therapeutic anticoagulation reduced the risk of thrombotic events with increased incidence of bleeding events in critically COVID-19 patients admitted to ICU. We recommended the use of prophylactic anticoagulation against empirical intermediate-to-therapeutic anticoagulation among unselected hospitalized COVID-19 patients based on low certainty in the evidence. For those with severer illness, the benefits to reduce thrombotic events of intermediate-to-therapeutic anticoagulation should also be weighed cautiously by clinicians because of the significantly increased risk of bleeding associated with higher dose of anticoagulant use. Furthermore, the optimal timing of anticoagulation initiation and treatment duration remains unexplored in this field. More well-designed RCTs that effectively stratify included patients by disease severity, timing of anticoagulation initiation, and treatment duration are highly encouraged to provide high-quality evidence regarding the safety and efficacy of higher dose anticoagulation in different clinical settings.

## Supplementary Information

**Additional file 1.** Detailed search strategy.

**Additional file 2.** Baseline characteristics of each study.

**Additional file 3.** Details of anticoagulation administration in each study.

**Additional file 4.** Quality assessment of cohort studies by NOS score.

**Additional file 5.** Quality assessment of RCTs by Cochrane Collaboration tool.

**Additional file 6.** Sensitivity analysis of in-hospital mortality outcome.

**Additional file 7.** Funnel plot for the assessment risk of publication bias of in-hospital mortality outcome.

**Additional file 8.** The quality of evidence for in-hospital mortality assessed by GRADE framework.

**Additional file 9.** Subgroup analysis of in-hospital mortality stratified by study regions.

**Additional file 10.** Sensitivity analysis of bleeding events outcome.

**Additional file 11.** Funnel plot for the assessment risk of publication bias of bleeding events outcome.

**Additional file 12.** The quality of evidence for bleeding events assessed by GRADE framework.

**Additional file 13.** Sensitivity analysis of thrombotic complication events outcome.

**Additional file 14.** Funnel plot for the assessment risk of publication bias of thrombotic complication events outcome.

**Additional file 15.** The quality of evidence for thrombotic complication events assessed by GRADE framework.

## Data Availability

The dataset generated and analyzed during the current study can be found in the included studies and their additional information files.

## Abbreviations

ICU: Intensive care unit
COVID-19: Coronavirus disease
RR: Risk ratio
RCT: Randomized clinical trail
CI: Confidence interval
UFH: Unfractionated heparin
LMWH: Low-molecular-weight heparin
I-TAC: Intermediate-to-therapeutic anticoagulation
PAC: Prophylactic anticoagulation
