# Supplementary material for: Intermediate-to-therapeutic versus prophylactic anticoagulation for coagulopathy in hospitalized COVID-19 patients: a systemic review and meta-analysis
Source: Thromb J. 2021 Nov 24;19:91. doi: 10.1186/s12959-021-00343-1 (PMC8611638; doi:10.1186/s12959-021-00343-1)
Supplement: Supplementary file 1 — Additional file 1. Detailed search strategy. [file 12959_2021_343_MOESM1_ESM.docx]

**Additional file 1. Detailed search strategy**

| #1 | COVID-19 |
| --- | --- |
| #2 | 2019 novel coronavirus infection |
| #3 | Coronavirus disease-2019 |
| #4 | 2019-nCoV disease |
| #5 | 2019 novel coronavirus disease |
| #6 | Severe acute respiratory syndrome coronavirus 2 |
| #7 | Wuhan coronavirus |
| #8 | Wuhan seafood market pneumonia virus |
| #9 | SARS-CoV-2 |
| #10 | SARS2 |
| #11 | #1 or #2 or #3 or #4 or #5 or #6 or #7 or #8 or #9 or #10 |
| #12 | Anticoagulant |
| #13 | Anticoagulation |
| #14 | Heparin |
| #15 | Unfractionated heparin |
| #16 | UFH |
| #17 | Fondaparinux |
| #18 | Enoxaparin |
| #19 | Low-molecular-weight heparin |
| #20 | “Heparin, Low Molecular Weight |
| #21 | LMWH |
| #22 | Thromboprophylaxis |
| #23 | Antithrombotic |
| #24 | Anti-thrombosis |
| #25 | #12 or #13 or #14 or #15 or #16 or #17 or #18 or #19 or #20 or #21 or #22 or #23 or #24 |
| #26 | #11 and #25 |
