# Supplementary material for: Intermediate-to-therapeutic versus prophylactic anticoagulation for coagulopathy in hospitalized COVID-19 patients: a systemic review and meta-analysis
Source: Thromb J. 2021 Nov 24;19:91. doi: 10.1186/s12959-021-00343-1 (PMC8611638; doi:10.1186/s12959-021-00343-1)
Supplement: Supplementary file 2 — Additional file 2. Baseline characteristics of each study. [file 12959_2021_343_MOESM2_ESM.docx]

**Additional file 2. Baseline characteristics of each study.**

| Author, publication year | Study location | Study design | Sample size(n) | Mean age(years) | Male (%) | Clinical setting | All-cause in-hospital mortality | | Incidence of bleeding events | |
| --- | --- | --- | --- | --- | --- | --- | --- | --- | --- | --- |
|  |  |  |  |  |  |  | I-TAC | PAC | I-TAC | PAC |
| Lemos et al 2020 | Brazil | RCT | 20 | 56.5 | 80 | Not reported | 20.00% | 50.00% | 60.00% | 20.00% |
| Bikdeli et al 2021 | Iran | RCT | 562 | 61.5 | 57.8 | ICU | 46.01% | 43.01% | 6.16% | 3.50% |
| Goligher et al 2021 | EU (international) | RCT | 1103 | 61.1 | 70 | ICU | 37.27% | 35.46% | 3.71% | 2.31% |
| Lopes et al 2021 | Brazil | RCT | 614 | 56.6 | 61 | Not reported | 11.29% | 7.57% | 8.39% | 2.30% |
| Lawler et al 2021 | EU (international) | RCT | 2231 | 58.9 | 58.7 | Non-ICU | 7.29% | 8.22% | 1.86% | 0.86% |
| Perepu et al 2021 | USA | RCT | 176 | 64 | 56 | ICU | 14.94% | 20.93% | 9.20% | 9.30% |
| Bolzetta et al 2020 | Italy | RC | 81 | 84.1 | 38.1 | Not reported | 50.00% | 50.88% | Not reported | |
| Canoglu et al 2020 | Turkey | RC | 154 | 60 | 62.3 | Mixed | 17.86% | 44.90% | Not reported | |
| Daughety et al 2020 | USA | RC | 126 | 61 | 63.3 | Mixed | 37.04% | 24.24% | 3.70% | 3.03% |
| Di Castelnuovo et al 2021 | Italy | RC | 1401 | 68 | 60.4 | Mixed | 14.83% | 11.60% | Not reported | |
| Elmelhat et al 2020 | United Arab Emirates | RC | 59 | 47.2 | 79.7 | Mixed | 7.69% | 2.50% | 7.69% | 2.50% |
| Ferguson et al 2020 | USA | RC | 141 | 64 | 55.3 | ICU | 26.09% | 29.47% | 26.09% | 8.42% |
| Halaby et al 2020 | USA | RC | 443 | 66 | 56.7 | ICU | Not reported | | Not reported | |
| Hanif et al 2020 | USA | RC | 863 | 62 | 62.3 | Not reported | 45.03% | 32.44% | Not reported | |
| Hsu et al 2020 | USA | RC | 441 | 64.5 | 54.9 | Not reported | 31.25% | 14.85% | 10.94% | 2.65% |
| Ionescu et al 2021 | USA | RC | 3480 | 64.5 | 48.5 | Mixed | 30.76% | 33.10% | 8.12% | 2.17% |
| Jean Francois et al 2020 | France | RC | 26 | 68 | 77 | ICU | 11.11% | 12.50% | Not reported | |
| Johnmarker et al 2020 | USA | RC | 152 | 61 | 82.2 | ICU | 20.00% | 38.81% | 9.41% | 11.94% |
| Helms et al 2021 | France | RC | 179 | 62 | 73 | ICU | 15.49% | 18.52% | 1.41% | 1.85% |
| Kaur et al 2020 | USA | RC | 1033 | 68 | 52.4 | Not reported | 28.61% | 20.25% | Not reported | |
| Kodama et al 2021 | USA | RC | 580 | 66 | 53 | Mixed | Not reported | | 9.76% | 3.61% |
| Lynn et al 2021 | England | RC | 502 | Not reported | 54.7 | Mixed | 34.87% | 15.20% | 8.55% | 3.20% |
| Marco et al 2021 | Italy | RC | 436 | 70.8 | 57.1 | Not reported | 26.85% | 25.44% | 9.52% | 4.62% |
| Martinelli et al 2021 | Italy | RC | 278 | 65.1 | 59 | ICU | 9.45% | 13.91% | 3.15% | 0.33% |
| Moll et al 2020 | USA | RC | 205 | 58.26 | 65.3 | ICU | 25.53% | 27.66% | 10.64% | 4.26% |
| Motta et al 2020 | USA | RC | 374 | 64.7 | 58.6 | Mixed | 38.67% | 14.38% | Not reported | |
| Meizlish et al 2021 | USA | RC | 382 | Not reported | 50.1 | Not reported | 9.95% | 19.37% | Not reported | |
| Musoke et al 2020 | USA | RC | 355 | 66.21 | 51 | Not reported | 41.58% | 15.28% | 9.84% | 3.93% |
| Nadkarni et al 2020 | USA | RC | 2859 | 65 | 56 | Not reported | 28.56% | 21.64% | 3.00% | 1.68% |
| Pablo et al 2021 | Spain | RC | 1965 | Not reported | Not reported | Mixed | Not reported | | 18.12% | 10.86% |
| Paolisso et al 2020 | Italy | RC | 510 | 65 | 63 | Not reported | 4.49% | 20.78% | Not reported | |
| Paranjpe et al 2020 | USA | RC | 2773 | Not reported | Not reported | Not reported | 22.77% | 22.40% | 3.05% | 1.91% |
| Pesavento et al 2020 | Italy | RC | 324 | 71.8 | 55.9 | Non-ICU | 16.67% | 11.25% | 21.43% | 6.25% |
| Poulakou et al 2021 | Greek | RC | 80 | 59.5 | 61.1 | Not reported | 3.70% | 3.85% | 3.70% | 3.85% |
| Qin et al 2021 | China | RC | 749 | 60 | 48 | Not reported | 32.47% | 17.43% | Not reported | |
| Nadeem et al 2021 | United Arab Emirates | RC | 149 | 50.7 | 86.6 | ICU | 54.76% | 67.65% | Not reported | |
| Rodolfo et al 2021 | Mexico | RC | 321 | 54 | 66.7 | Not reported | 5.66% | 8.26% | 7.08% | 6.42% |
| Takayama et al 2021 | Japan | RC | 62 | 57 | 87.1 | ICU | 0.00% | 17.24% | Not reported | |
| Vaughn et al 2021 | USA | RC | 1351 | 64 | 52.3 | Not reported | 26.85% | 21.84% | Not reported | |
| Yu et al 2021 | USA | RC | 348 | 63.9 | 63.6 | Not reported | 60.15% | 60.93% | 13.53% | 3.72% |
| Longhitano et al 2020 | Italy | PC | 74 | 68.65 | 59.5 | Mixed | 21.28% | 7.41% | Not reported | |
| Voicu et al 2021 | France | PC | 93 | 63 | 69 | ICU | 46.51% | 36.00% | 25.58% | 14.00% |

RCT: Randomized Controlled Trial; RC: Retrospective Cohort; PC: Prospective Cohort.
