# Supplementary material for: Intermediate-to-therapeutic versus prophylactic anticoagulation for coagulopathy in hospitalized COVID-19 patients: a systemic review and meta-analysis
Source: Thromb J. 2021 Nov 24;19:91. doi: 10.1186/s12959-021-00343-1 (PMC8611638; doi:10.1186/s12959-021-00343-1)
Supplement: Supplementary file 3 — Additional file 3. Details of anticoagulation administration in each study. [file 12959_2021_343_MOESM3_ESM.docx]

**Additional file 3. Details of anticoagulant administration in each study**

| **Author, publication year** | **I-TAC** | **PAC** |
| --- | --- | --- |
| **Lemos et al, 2020** | 1. Subcutaneous enoxaparin: a) Patients under 75-year-old with CrCl>50 mL/min received 1 mg/Kg bid; b) with CrCl between 30 and 50 mL/min: 0.75 mg/Kg bid; c) with CrCl between 10 and 30 mL/min: 1 mg/Kg once daily; d) with CrCl<10 mL/min: intravenous UFH 24h after the last dose of enoxaparin. 2. UFH: a) Patients older than 75 years with CrCl>50 mL/min received: 0.75 mg/Kg bid; 2) with CrCl between 30 and 50 mL/min: 1 mg/Kg once daily; 3) with CrCl between 10 and 30 mL/min: 0.75 mg/Kg once daily; 4) with CrCl<10 mL/min: similar to younger patients. | 1. Subcutaneous UFH at a dose of 5000 IU tid (if weight < 120 kg); 2. Subcutaneous UFH at a dose of 7500 IU tid (if weight > 120 kg) 3. Enoxaparin at a dose of 40 mg once daily (if weight < 120 kg) and 40 mg bid (if weight > 120 Kg) according to the doctor's judgment. |
| **Bikdeli et al, 2021** | 1. Heparin-based regimen; 2. For patients who weighed <120 kg and had a creatinine clearance >30mL/min, enoxaparin 1mg/kg once daily. | Enoxaparin 40mg once daily |
| **Goligher et al, 2021** | Not reported | Not reported |
| **Lopes et al, 2021** | 1. Oral rivaroxaban (20 mg or 15 mg daily) for stable patients; 2. Initial subcutaneous enoxaparin (1 mg/kg twice per day); 3. Intravenous UFH (to achieve a 0·3–0·7 IU/mL anti-Xa concentration) for clinically unstable patients, followed by rivaroxaban to day 30 | Not reported |
| **Lawler et al, 2021** | Not reported | Not reported |
| **Perepu et al, 2021** | 1. 1 mg/kg enoxaparin daily (BMI< 30); 2. 0.5 mg/kg enoxaparin twice daily (BMI ≥ 30) | 1. 40 mg enoxaparin daily (BMI<30) and either 30 mg enoxaparin twice daily; 2. 40 mg enoxaparin twice daily (BMI ≥ 30) |
| **Bolzetta et al, 2020** | Not reported | Not reported |
| **Canoglu et al, 2020** | 1 mg/kg LMWH twice daily. | 0.5 mg/kg LMWH twice daily. |
| **Daughety et al, 2020** | 1. Enoxaparin 0.5 mg/kg twice daily; 2. Heparin infusion titrated to anti-factor Xa levels 0.3–0.5 U/mL in patients with renal failure (CrCl < 30 mL/min) | 1. Enoxaparin 40 mg daily if weight <100 kg and 60 mg daily if weight >100 kg; 2. 5000 U of UFH three times daily in patients with renal failure. |
| **Di Castelnuovo et al, 2021** | Not reported | Not reported |
| **Elmelhat et al, 2020** | 1 mg/kg enoxaparin twice per day. | 40 mg enoxaparin once per day. |
| **Ferguson et al, 2020** | 1. Continuous infusion of heparin dose-adjusted based on UFH level or by subcutaneous 1 mg/kg twice daily; 2. 1.5 mg/kg daily LMWH (LMWH dose adjustments were made based on anti-Xa levels in the event of renal insufficiency). | 1. Enoxaparin 40 mg subcutaneously daily; 2. Enoxaparin 30 mg twice daily; 3. Enoxaparin 0.5 mg/kg twice daily; 4. Heparin 5000 units subcutaneously 2 or 3 times daily. |
| **Halaby et al, 2020** | 1. Enoxaparin 0.5 mg/kg twice daily; 2. Enoxaparin 40 mg twice daily for patients with BMI <40 kg/m2; 3. Subcutaneous heparin 7500 units every 8 h; 4. UFH drip adjusted by partial thromboplastin time or anti‐XA level, argatroban infusion, bivalirudin infusion, enoxaparin 1 mg/kg twice a day or 1.5 mg/kg daily; 5. Fondaparinux ≥5 mg daily; 6. Warfarin, apixaban 5 or 10 mg twice daily; 7. Rivaroxaban 15 mg twice daily or 20 mg once daily; 8. Dabigatran 150 mg twice daily. | 1. Enoxaparin ≤40 mg once daily; 2. Enoxaparin 40 mg twice daily if body mass index (BMI) ≥ 40 kg/m2; 3. Subcutaneous heparin 5000 units or less 2 or 3 times a day; 4. Apixaban 2.5 mg twice daily (with intent of prophylaxis); 5. Rivaroxaban 10 mg daily; 6. Betrixaban 80 or 160 mg once daily; heparin infusion 200–500 units/h without partial thromboplastin time titration (used for continuous renal replacement therapy); 7. Fondaparinux 2.5 mg once daily. |
| **Hanif et al, 2020** | Not reported | Not reported |
| **Hsu et al, 2020** | 1. LMWH 40 mg twice daily; 2. UFH subcutaneous 7500 units three times daily | 1. LMWH 40 mg once daily, UFH subcutaneous 5000 units three times daily; 2. apixaban 2.5 mg twice daily |
| **Ionescu et al, 2021** | 1. Intravenous UFH with at least one documented activated partial thromboplastin time in the anticoagulation range (≥45 seconds); 2. Subcutaneous enoxaparin at doses of 1 mg/kg twice daily or 1.5 mg/kg once daily (while allowing for dose adjustment based on creatinine clearance); 3. Intravenous argatroban infusion; 4. Subcutaneous fondaparinux at doses of 5‐10 mg once daily (weight‐based dosing); 5. Oral anticoagulants (warfarin, apixaban, rivaroxaban, dabigatran) prescribed prior to and continued throughout hospitalization | 1. Subcutaneous injection of UFH at doses of 5000 units twice or three times daily; 2. Subcutaneous enoxaparin injection at doses of 30‐40 mg once daily; 3. Subcutaneous fondaparinux at a dose of 2.5 mg once daily |
| **Jean Francois et al, 2020** | 1. Either low molecular weight heparin or UFH with anti‐Xa monitoring, with therapeutic levels of 0.3 to 0.7 U/mL of anti‐Xa activity 2. Anticoagulation dose was left to the discretion of the treating physician based on the individual risk of thrombosis | The discretion of the treating physician based on the individual risk of thrombosis |
| **Johnmarker et al, 2020** | 1. ≥ 175 IU/kg of body weight tinzaparin; 2. ≥ 200 IU/kg of body weight dalteparin; 3. 4500 IU but < 175 IU/kilogram, kg, of body weight tinzaparin; 4. > 5000 IU but < 200 IU/kg of body weight dalteparin. | 1. 2500–4500 IU tinzaparin; 2. 2500–5000 IU dalteparin |
| **Helms et al, 2021** | LMWH 100 IU/kg/12h, without exceeding 10000 IU/12h or UFH 500 IU/kg/24h | LMWH up to 6000 IU/12h or UFH 200 IU/kg/24h |
| **Kaur et al, 2020** | Not reported | Not reported |
| **Kodama et al, 2021** | Not reported | Not reported |
| **Lynn et al, 2021** | Not reported | Not reported |
| **Marco et al, 2021** | > 4000 IU daily dosage of enoxaparin | 4000 IU daily dosage of enoxaparin |
| **Martinelli et al, 2021** | 1. Patients in the ICU received 1 mg/kg twice daily; 2. Patients in high-intensity of care wards 0.7 mg/kg twice daily; 3. Patients in low-intensity of care wards 1 mg/kg daily | Enoxaparin 40 mg daily increased to 60 mg daily in obese |
| **Moll et al, 2020** | 1. Enoxaparin 40 mg twice daily, adjusted for extremes of weight (0.5 mg/kg twice daily) 2. 7500 IU UFH three times daily | 1. Enoxaparin 40 mg daily; 2. UFH 5000 IU twice or three times daily |
| **Motta et al, 2020** | 1. Enoxaparin: 1 mg/kg subcutaneously bid or 1.5 mg/kg subcutaneously daily or based on renal function, or higher doses titrated to anti-Factor Xa range of 0.6–1 IU/mL (for bid dosing) and 1–2 IU/mL (for daily dosing); 2. Heparin: IV heparin titrated to an activated partial thromboplastin time between 70 and 110 s | 1. Enoxaparin: 30 or 40 mg subcutaneously every day; 2. Heparin: 5,000 units given subcutaneously every 8 hours |
| **Meizlish et al, 2021** | 1. Maximum enoxaparin dose ≥0.4 and < 0.7 mg/kg every 12 h 2. Subcutaneous UFH 7500 U at any frequency with a BMI < 40, and who did not receive any other type of anticoagulant during their hospitalization; 3. Maximum enoxaparin dose ≥0.7 mg/kg every 12 h; 4. Enoxaparin ≥0.7 mg/kg every 24 h with creatinine clearance < 30 mL/min; 5. Enoxaparin ≥1.4 mg/kg every 24 h | 1. Maximum enoxaparin dose of 30–40 mg at a weight-adjusted concentration of < 0.7 mg/kg every 24 h; 2. Enoxaparin 30–40 mg at a weight-adjusted concentration of < 0.4 mg/kg every 12 h; 3. Subcutaneous UFH 5000 units up to three times per day, 4. Subcutaneous UFH 5000 or 7500 units up to three times per day with a BMI ≥40 kg/m2, and who did not receive any other type of documented anticoagulant during their hospitalization |
| **Musoke et al, 2020** | 1 mg/kg LMWH q12h | 1. Heparin 5000 units subcutaneously 2–3 times/day; 2. LMWH 30-40 mg daily. |
| **Nadkarni et al, 2020** | 1. Bivalirudin, argatroban or UFH, high dose LMWH (specifically enoxaparin 1 mg/kg twice daily or 1.5 mg/kg daily); 2. Apixaban 5mg twice daily | 1. Apixaban at 2.5 mg twice a day or 5 mg once a day (patients >75 years); 2. Subcutaneous UFH, LMWH once daily; 3. Apixaban (2.5 mg twice a day or 5 mg daily in patients ≤75 years) |
| **Pablo et al, 2021** | 1. Enoxaparin 120 mg; 2. Enoxaparin 160 mg; 3. Enoxaparin 200 mg; 4. Biosimilars enoxaparin 120 mg; 5. Biosimilars enoxaparin 160 mg; 6. Bemiparin 10 000–12500 IU; 7. Tinzaparin 10 000–14000 IU; 8. Edoxaban 60 mg; 9. Rivaroxaban 20 mg; 10. Dabigatran 220 mg | 1. Enoxaparin 60 mg; 2. Enoxaparin 80 mg; 3. Enoxaparin 100 mg; 4. Bemiparin 5000–7500IU; 5. Biosimilars enoxaparin 60 mg; 6. Biosimilars enoxaparin 80 mg; 7. Biosimilars enoxaparin 100 mg; 8. Apixaban 5 mg; 9. Edoxaban 30 mg; 10. Rivaroxaban 10 mg; 11. Rivaroxaban 15 mg |
| **Paolisso et al, 2020** | Subcutaneous enoxaparin 40–60 mg twice daily | Subcutaneous enoxaparin 40–60 mg daily |
| **Paranjpe et al, 2020** | Not reported | Not reported |
| **Pesavento et al, 2020** | 1. LMWH 1 mg/kg bid; 2. LMWH 0.5 mg/kg bid; 3. Fondaparinux 7.5 mg | 1. UFH infusion 5000 U tid; 2. LMWH 40 mg once daily; 3. Fondaparinux 2.5 mg |
| **Poulakou et al, 2021** | Not reported | Not reported |
| **Qin et al, 2021** | 100 U/kg LMWH, q12h | 3000–5000 U/day LMWH |
| **Nadeem et al, 2021** | Not reported | Not reported |
| **Rodolfo et al, 2021** | 1) 0.5 mg/kg enoxaparin of weight bid or 40 mg bid;  2) 1 mg/kg enoxaparin of weight bid. | 40 mg of enoxaparin qd |
| **Takayama et al, 2021** | UFH 4,850 unit/kg | UFH 1,930 unit/kg |
| **Vaughn et al, 2021** | 1. Apixaban (Eliquis), dose unknown; 2. Edoxavan (Savaysa) , dose unknown; 3. Enoxaparin (Lovenox) , dose unknown; 4. Heparin (Intravenous) , dose unknown; 5. Rivaroxaban (Xarelto) , dose unknown; 6. Warfarin (Coumadin) , dose unknown | 1) Enoxaparin (Lovenox) 30-40 mg daily;  2) Enoxaparin (Lovenox) 30-40 mg bid;  3) Fondaparinux (Arixtra) 2.5 mg Daily;  4) Heparin ≤15,000 daily units;  5) Heparin >15,000 daily units;  6) Apixaban (with prophylactic intent);  7) Intravenous Heparin (with prophylactic intent) |
| **Yu et al, 2021** | Enoxaparin (1mg/kg twice daily), apixaban (≥ 5 mg twice daily), UFH infusion, and fondaparinux (≥ 5 mg once daily). | unknown |
| **Longhitano et al, 2020** | 1. Enoxaparin<200, >80U/kg/qd; 2. Heparin>15000, <25000U/qd; 3. Fondaparinux 5mg/24h; 4. Enoxaparin 100U/kg bid; 5. Heparin 12500U bid/tid | 1. Enoxaparin 80U/kg/qd; 2. Heparin 5000 U tid; 3. Fondaparinux 2.5mg/qd |
| **Voicu et al, 2021** | 1. Prophylactic double-dose enoxaparin 40 mg twice daily; 2. Therapeutic anticoagulation with either enoxaparin 1 mg/kg twice daily; 3. UFH to reach plasma anti-Xa activity of 0.3–0.6IU/mL. | 1. Subcutaneous enoxaparin 40 mg once daily; 2. UFH 150 0 0IU/day if creatinine clearance < 15 mL/min. |

I-TAC: intermediate-therapeutic-dose anticoagulation; PAC: prophylactic-dose anticoagulation; LMWH: low-molecular-weight heparin; UFH: Unfractionated heparin
