# Supplementary material for: Intermediate-to-therapeutic versus prophylactic anticoagulation for coagulopathy in hospitalized COVID-19 patients: a systemic review and meta-analysis
Source: Thromb J. 2021 Nov 24;19:91. doi: 10.1186/s12959-021-00343-1 (PMC8611638; doi:10.1186/s12959-021-00343-1)
Supplement: Supplementary file 4 — Additional file 4. Quality assessment of cohort studies by NOS score. [file 12959_2021_343_MOESM4_ESM.docx]

**Additional file 4. Quality assessment of cohort studies by NOS score**

| Author, publication year | Study design | Selection | Comparability | Outcome | Final score |
| --- | --- | --- | --- | --- | --- |
| Bolzetta et al, 2020 | Cohort | **** | ** | ** | 8 |
| Canoglu et al, 2020 | Cohort | **** | ** | *** | 9 |
| Daughety et al, 2020 | Cohort | **** | ** | ** | 8 |
| Di Castelnuovo et al, 2021 | Cohort | **** | ** | *** | 9 |
| Elmelhat et al, 2020 | Cohort | *** | ** | ** | 7 |
| Ferguson et al, 2020 | Cohort | *** | ** | *** | 8 |
| Halaby et al, 2020 | Cohort | **** | ** | *** | 9 |
| Hanif et al, 2020 | Cohort | **** | ** | *** | 9 |
| Hsu et al, 2020 | Cohort | *** | ** | ** | 7 |
| Ionescu et al, 2020 | Cohort | *** | ** | *** | 8 |
| Johnmarker et al, 2020 | Cohort | *** | ** | *** | 8 |
| Helms et al, 2021 | Cohort | **** | ** | *** | 9 |
| Longhitano et al, 2020 | Cohort | *** | ** | *** | 8 |
| Lynn et al, 2021 | Cohort | *** | ** | *** | 8 |
| Marco et al, 2021 | Cohort | **** | ** | *** | 9 |
| Martinelli et al, 2021 | Cohort | *** | ** | *** | 8 |
| Meizlish et al, 2021 | Cohort | **** | ** | *** | 9 |
| Musoke et al, 2020 | Cohort | *** | ** | *** | 8 |
| Moll et al, 2021 | Cohort | **** | ** | *** | 9 |
| Pablo et al, 2021 | Cohort | **** | ** | *** | 9 |
| Paolisso et al, 2020 | Cohort | *** | ** | ** | 7 |
| Pesavento et al, 2020 | Cohort | *** | ** | *** | 8 |
| Poulakou et al, 2021 | Cohort | *** | ** | ** | 7 |
| Qin et al, 2021 | Cohort | *** | ** | *** | 8 |
| Nadeem et al, 2021 | Cohort | *** | ** | *** | 8 |
| Rodolfo et al, 2021 | Cohort | *** | ** | *** | 8 |
| Nadkarni et al, 2021 | Cohort | *** | ** | *** | 8 |
| Jean Francois et al, 2021 | Cohort | *** | ** | *** | 8 |
| Voicu et al, 2021 | Cohort | *** | ** | *** | 8 |
| Paranjpe, 2020 | Cohort | *** | ** | *** | 8 |
| Takayama et al, 2021 | Cohort | **** | ** | *** | 9 |
| Vaughn et al, 2021 | Cohort | *** | ** | *** | 8 |
| Yu et al, 2021 | Cohort | **** | ** | *** | 9 |
| Motta et al, 2020 | Cohort | *** | ** | *** | 8 |
| Kodama et al, 2021 | Cohort | ** | ** | ** | 6 |
| Kaur et al, 2020 | Cohort | *** | ** | *** | 8 |
