# Supplementary figures and images for: Intermediate-to-therapeutic versus prophylactic anticoagulation for coagulopathy in hospitalized COVID-19 patients: a systemic review and meta-analysis
Source: Thromb J. 2021 Nov 24;19:91. doi: 10.1186/s12959-021-00343-1 (PMC8611638; doi:10.1186/s12959-021-00343-1)

**Additional file 5. Quality assessment of RCTs by Cochrane Collaboration tool**

**
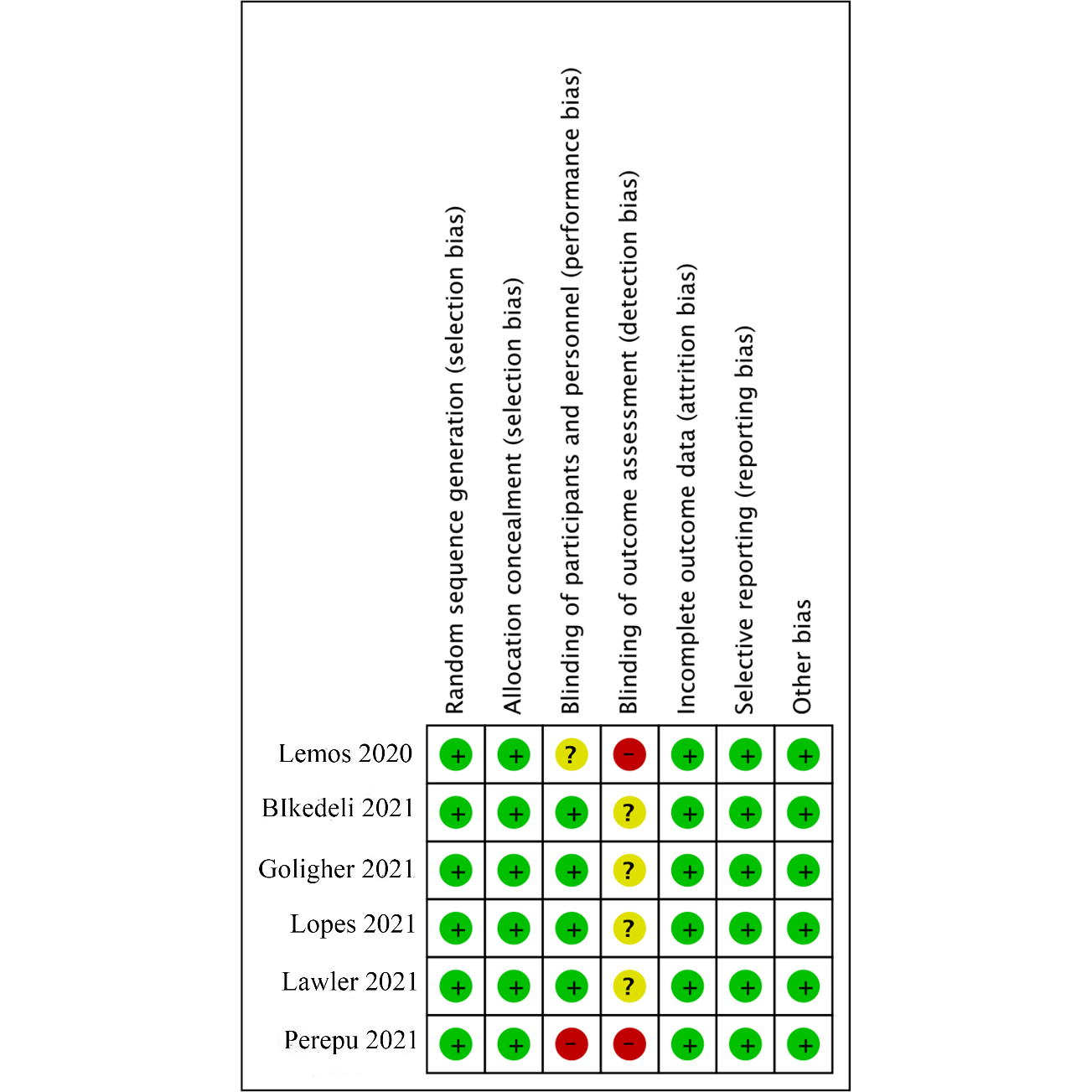
**

Supplement: Supplementary file 5 — Additional file 5. Quality assessment of RCTs by Cochrane Collaboration tool. [file 12959_2021_343_MOESM5_ESM.docx]

**Additional file 9. Subgroup analysis of in-hospital mortality stratified by study regions (.tif)**

**
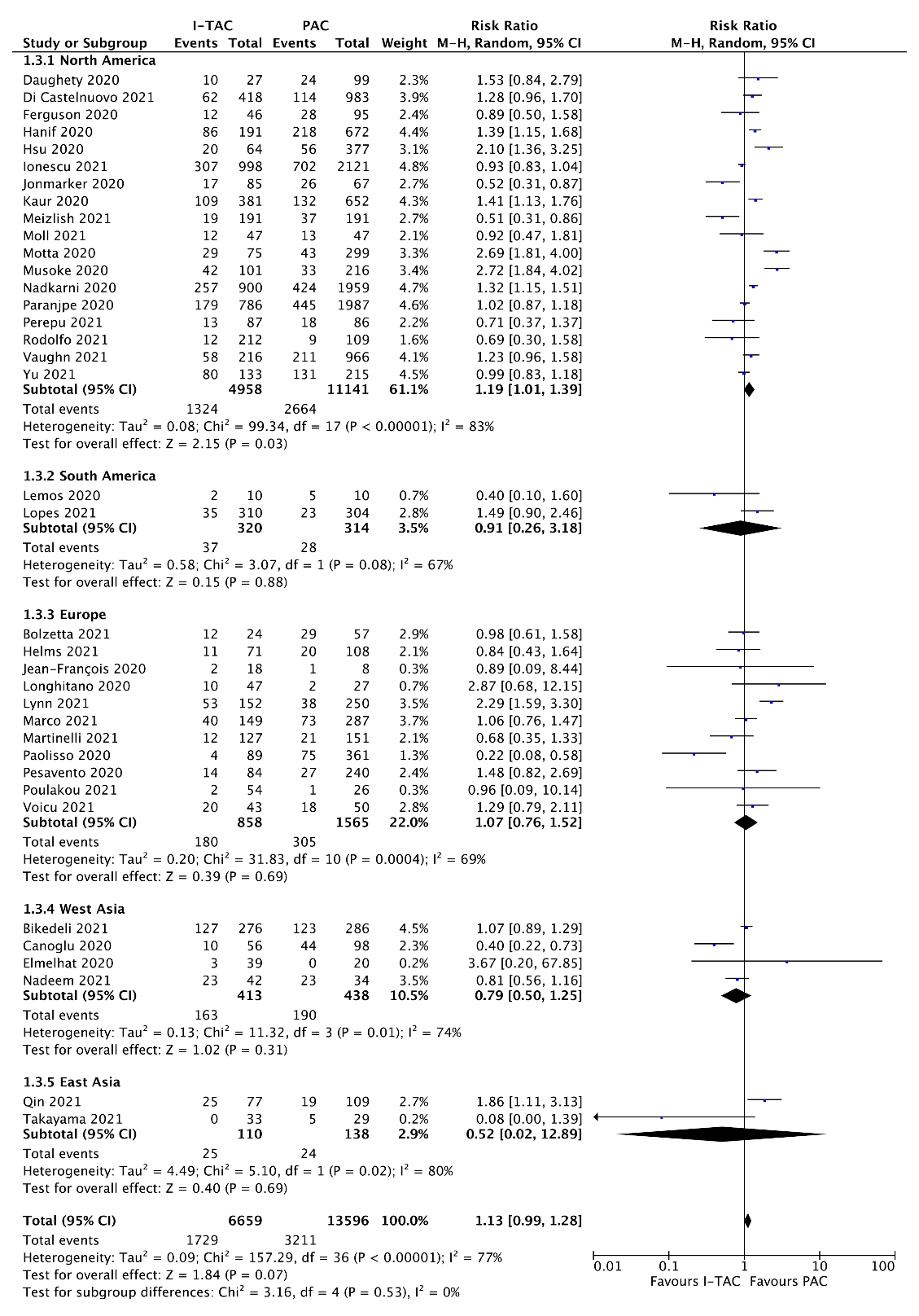
**

Supplement: Supplementary file 9 — Additional file 9. Subgroup analysis of in-hospital mortality stratified by study regions. [file 12959_2021_343_MOESM9_ESM.docx]
