# Supplementary material for: Intermediate-to-therapeutic versus prophylactic anticoagulation for coagulopathy in hospitalized COVID-19 patients: a systemic review and meta-analysis
Source: Thromb J. 2021 Nov 24;19:91. doi: 10.1186/s12959-021-00343-1 (PMC8611638; doi:10.1186/s12959-021-00343-1)
Supplement: Supplementary file 6 — Additional file 6. Sensitivity analysis of in-hospital mortality outcome. [file 12959_2021_343_MOESM6_ESM.docx]

**Additional file 6. Sensitivity analysis of in-hospital mortality outcome**

| Omitting study | RR [95%-CI] | p value |
| --- | --- | --- |
| Lemos et al, 2020 | 1.1239 [1.0001;1.2629] | 0.0498 |
| Bikedeli et al, 2021 | 1.1164 [0.9877;1.2617] | 0.0781 |
| Bolzetta et al, 2020 | 1.1200 [0.9944;1.2614] | 0.0618 |
| Canoglu et al, 2020 | 1.1432 [1.0194;1.2819] | 0.0221 |
| Daughety et al, 2020 | 1.1089 [0.9854;1.2480] | 0.0862 |
| Di Castelnuovo et al, 2021 | 1.1099 [0.9843;1.2516] | 0.0888 |
| Elmelhat et al, 2020 | 1.1147 [0.9917;1.2529] | 0.0686 |
| Goligher et al, 2021 | 1.1166 [0.9869;1.2634] | 0.0801 |
| Ferguson et al, 2020 | 1.1221 [0.9969;1.2631] | 0.0564 |
| Hanif et al, 2020 | 1.1052 [0.9797;1.2466] | 0.1037 |
| Hsu et al, 2020 | 1.0974 [0.9768;1.2329] | 0.1175 |
| Ionescu et al, 2020 | 1.1237 [0.9939;1.2705] | 0.0626 |
| Jean-François et al, 2020 | 1.1170 [0.9936;1.2558] | 0.0640 |
| Jonmarker et al, 2020 | 1.1396 [1.0149;1.2796] | 0.0271 |
| Helms et al, 2021 | 1.1224 [0.9975;1.2630] | 0.0551 |
| Kaur et al, 2020 | 1.1049 [0.9800;1.2458] | 0.1030 |
| Longhitano et al, 2020 | 1.1106 [0.9882;1.2482] | 0.0783 |
| Lopes et al, 2021 | 1.1080 [0.9842;1.2473] | 0.0897 |
| Lynn et al, 2021 | 1.0928 [0.9746;1.2253] | 0.1285 |
| Marco et al, 2021 | 1.1179 [0.9916;1.2604] | 0.0684 |
| Martinelli et al, 2021 | 1.1272 [1.0022;1.2678] | 0.0459 |
| Moll et al, 2021 | 1.1203 [0.9954;1.2608] | 0.0596 |
| Motta et al, 2020 | 1.0901 [0.9738;1.2203] | 0.1339 |
| Meizlish et al, 2021 | 1.1400 [1.0154;1.2799] | 0.0266 |
| Musoke et al, 2020 | 1.0895 [0.9737;1.2191] | 0.1350 |
| Nadkarni et al, 2021 | 1.1056 [0.9778;1.2502] | 0.1093 |
| Paolisso et al, 2020 | 1.1385 [1.0158;1.2759] | 0.0257 |
| Paranjpe et al, 2020 | 1.1183 [0.9885;1.2652] | 0.0756 |
| Lawler et al, 2021 | 1.1259 [0.9991;1.2689] | 0.0517 |
| Perepu et al, 2021 | 1.1264 [.0013;1.2671] | 0.0475 |
| Pesavento et al, 2020 | 1.1095 [0.9858;1.2487] | 0.0849 |
| Poulakou et al, 2021 | 1.1167 [0.9933;1.2555] | 0.0646 |
| Qin et al, 2021 | 1.1028 [0.9805;1.2403] | 0.1028 |
| Nadeem et al, 2021 | 1.1286 [1.0023;1.2709] | 0.0457 |
| Rodolfo et al, 2021 | 1.1242 [0.9996;1.2644] | 0.0507 |
| Voicu et al, 2021 | 1.1118 [0.9873;1.2521] | 0.0804 |
| Takayama et al, 2021 | 1.1215 [0.9987;1.2595] | 0.0527 |
| Vaughn et al, 2021 | 1.1110 [0.9847;1.2536] | 0.0874 |
| Yu et al, 2021 | 1.1205 [0.9916;1.2662] | 0.0681 |
