# Supplementary material for: Intermediate-to-therapeutic versus prophylactic anticoagulation for coagulopathy in hospitalized COVID-19 patients: a systemic review and meta-analysis
Source: Thromb J. 2021 Nov 24;19:91. doi: 10.1186/s12959-021-00343-1 (PMC8611638; doi:10.1186/s12959-021-00343-1)

**Additional file 7. Funnel plot for the assessment risk of publication bias of in-hospital mortality outcome**


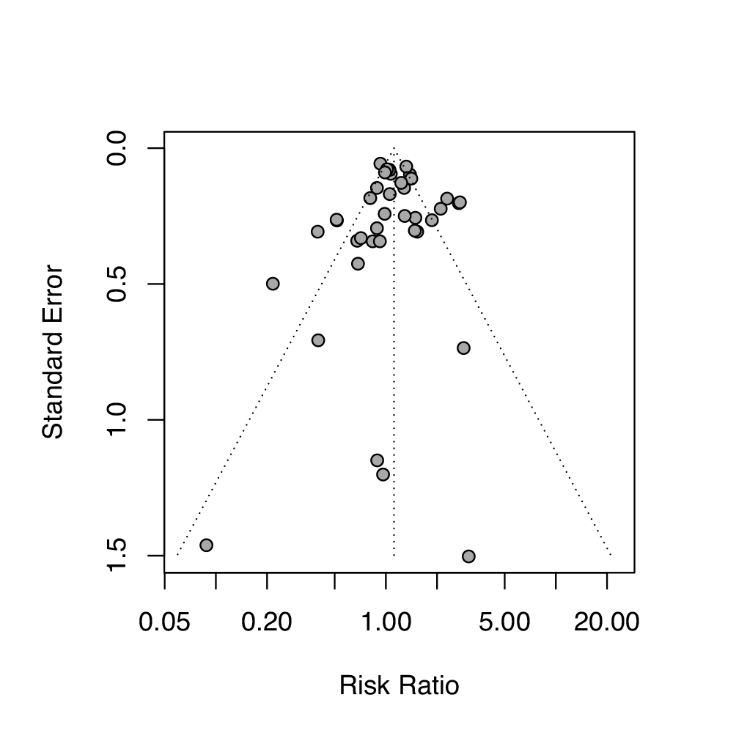

Supplement: Supplementary file 7 — Additional file 7. Funnel plot for the assessment risk of publication bias of in-hospital mortality outcome. [file 12959_2021_343_MOESM7_ESM.docx]
