# Supplementary material for: Intermediate-to-therapeutic versus prophylactic anticoagulation for coagulopathy in hospitalized COVID-19 patients: a systemic review and meta-analysis
Source: Thromb J. 2021 Nov 24;19:91. doi: 10.1186/s12959-021-00343-1 (PMC8611638; doi:10.1186/s12959-021-00343-1)
Supplement: Supplementary file 8 — Additional file 8. The quality of evidence for in-hospital mortality assessed by GRADE framework. [file 12959_2021_343_MOESM8_ESM.docx]

**Additional file 8. The quality of evidence for in-hospital mortality assessed by GRADE framework**

| **Certainty assessment** | | | | | | | **No. of patients** | | **Effect** | | **Certainty** | **Importance** |
| --- | --- | --- | --- | --- | --- | --- | --- | --- | --- | --- | --- | --- |
| **No. of studies** | **Study design** | **Risk of bias** | **Inconsistency** | **Indirectness** | **Imprecision** | **Other considerations** | **intermediate-to-therapeutic dose AC** | **prophylactic dose AC** | **Relative (95% CI)** | **Absolute (95% CI)** |  |  |
| **In-hospital mortality** | | | | | | | | | | | | |
| 39 | observational studies and  RCTs | serious | serious | not serious | not serious | none | 2014/8373 (24.1%) | 3497/15206 (23.0%) | **RR 1.12** (0.99 to 1.25) | **28 more per 1,000** (from 2 fewer to 57 more) | ⨁◯◯◯ VERY LOW | CRITICAL |
| **In-hospital mortality of critically ill patients admitted to ICU** | | | | | | | | | | | | |
| 14 | observational studies and  RCTs | not serious | not serious | not serious | not serious | all plausible residual confounding would reduce the demonstrated effect | 443/1480 (29.9%) | 524/1626 (32.2%) | **RR 0.82** (0.66 to 1.03) | **58 fewer per 1,000** (from 110 fewer to 10 more) | ⨁⨁⨁◯ MODERATE | CRITICAL |
