# Supplementary material for: Intermediate-to-therapeutic versus prophylactic anticoagulation for coagulopathy in hospitalized COVID-19 patients: a systemic review and meta-analysis
Source: Thromb J. 2021 Nov 24;19:91. doi: 10.1186/s12959-021-00343-1 (PMC8611638; doi:10.1186/s12959-021-00343-1)
Supplement: Supplementary file 10 — Additional file 10. Sensitivity analysis of bleeding events outcome. [file 12959_2021_343_MOESM10_ESM.docx]

**Additional file 10. Sensitivity analysis of bleeding events outcome**

| Omitting Study | RR [95%-CI] | p value |
| --- | --- | --- |
| Bikedeli et al, 2021 | 2.1746 [1.7933;2.6370] | <0.0001 |
| Daughety et al, 2020 | 2.1636 [1.7928;2.6109] | <0.0001 |
| Elmelhat et al, 2020 | 2.1522 [1.7831;2.5976] | <0.0001 |
| Goligher et al, 2021 | 2.1882 [1.8050;2.6528] | <0.0001 |
| Ferguson et al, 2020 | 2.1245 [1.7569;2.5692] | <0.0001 |
| Hsu et al, 2020 | 2.111 [1.7539;2.5408] | <0.0001 |
| Ionescu et al, 2020 | 1.9436 [1.6757;2.2545] | <0.0001 |
| Jonmarker et al, 2020 | 2.222 [1.8611;2.6530] | <0.0001 |
| Helms et al, 2021 | 2.1685 [1.7996;2.6129] | <0.0001 |
| Kodama et al, 2021 | 2.1352 [1.7625;2.5867] | <0.0001 |
| Lopes et al, 2021 | 2.1112 [1.7510;2.5456] | <0.0001 |
| Lynn et al, 2021 | 2.138 [1.7654;2.5892] | <0.0001 |
| Marco et al, 2021 | 2.1562 [1.7847;2.6052] | <0.0001 |
| Martinelli et al, 2021 | 2.1428 [1.7806;2.5788] | <0.0001 |
| Moll et al, 2021 | 2.1511 [1.7802;2.5992] | <0.0001 |
| Musoke et al, 2020 | 2.1442 [1.7703;2.5970] | <0.0001 |
| Nadkarni et al, 2021 | 2.1864 [1.7959;2.6620] | <0.0001 |
| Pablo et al, 2021 | 2.2395 [1.8482;2.7138] | <0.0001 |
| Paranjpe et al, 2020 | 2.2048 [1.8155;2.6776] | <0.0001 |
| Lawler et al, 2021 | 2.1546 [1.7759;2.6140] | <0.0001 |
| Perepu et al, 2021 | 2.2085 [1.8380;2.6538] | <0.0001 |
| Pesavento et al, 2020 | 2.0992 [1.7402;2.5322] | <0.0001 |
| Poulakou et al, 2021 | 2.1658 [1.7959;2.6119] | <0.0001 |
| Rodolfo et al, 2021 | 2.2075 [1.8339;2.6573] | <0.0001 |
| Voicu et al, 2021 | 2.1686 [1.7895;2.6280] | <0.0001 |
| Yu et al, 2021 | 2.1101 [1.7501;2.5441] | <0.0001 |
| Lemos et al, 2020 | 2.1427 [1.7735;2.5888] | <0.0001 |
