# Supplementary material for: Intermediate-to-therapeutic versus prophylactic anticoagulation for coagulopathy in hospitalized COVID-19 patients: a systemic review and meta-analysis
Source: Thromb J. 2021 Nov 24;19:91. doi: 10.1186/s12959-021-00343-1 (PMC8611638; doi:10.1186/s12959-021-00343-1)
Supplement: Supplementary file 12 — Additional file 12. The quality of evidence for bleeding events assessed by GRADE framework. [file 12959_2021_343_MOESM12_ESM.docx]

**Additional file 12 The quality of evidence for bleeding events assessed by GRADE framework**

| **Certainty assessment** | | | | | | | **No. of patients** | | **Effect** | | **Certainty** | **Importance** |
| --- | --- | --- | --- | --- | --- | --- | --- | --- | --- | --- | --- | --- |
| **No. of studies** | **Study design** | **Risk of bias** | **Inconsistency** | **Indirectness** | **Imprecision** | **Other considerations** | **intermediate-to-therapeutic dose AC** | **prophylactic dose AC** | **Relative (95% CI)** | **Absolute (95% CI)** |  |  |
| **Any bleeding events** | | | | | | | | | | | | |
| 27 | observational studies and  randomized  studies | not serious | not serious | not serious | not serious | strong association | 483/7113 (6.8%) | 405/12172 (3.3%) | **RR 2.16** (1.79 to 2.60) | **39 more per 1,000** (from 26 more to 53 more) | ⨁⨁⨁◯ MODERATE | CRITICAL |
| **Any bleeding events in critically ill patients admitted to ICU** | | | | | | | | | | | | |
| 10 | observational studies and  randomized  studies | not serious | not serious | not serious | not serious | none | 198/1939 (10.2%) | 190/2667 (7.1%) | **RR 1.66** (1.37 to 2.00) | **48 more per 1,000** (from 27 more to 72 more) | ⨁⨁◯◯ LOW | CRITICAL |
| **Major bleeding events** | | | | | | | | | | | | |
| 20 | observational studies and  randomized studies | not serious | not serious | not serious | not serious | strong association | 346/6906 (5.0%) | 297/12035 (2.5%) | **RR 2.11** (1.77 to 2.51) | **27 more per 1,000** (from 19 more to 37 more) | ⨁⨁⨁◯ MODERATE | CRITICAL |
