# Supplementary material for: Intermediate-to-therapeutic versus prophylactic anticoagulation for coagulopathy in hospitalized COVID-19 patients: a systemic review and meta-analysis
Source: Thromb J. 2021 Nov 24;19:91. doi: 10.1186/s12959-021-00343-1 (PMC8611638; doi:10.1186/s12959-021-00343-1)
Supplement: Supplementary file 13 — Additional file 13. Sensitivity analysis of thrombotic complication events outcome. [file 12959_2021_343_MOESM13_ESM.docx]

**Additional file 13. Sensitivity analysis of thrombotic complication events outcome**

| Omitting study | RR [95%-CI] | p value |
| --- | --- | --- |
| Lemos et al, 2020 | 1.3185 [0.7879;2.2065] | 0.2926 |
| Bikedeliv et al, 2021 | 1.3321 [0.7866;2.2561] | 0.2860 |
| Goligher et al, 2021 | 1.3730 [0.7972;2.3648] | 0.2531 |
| Hsu et al, 2020 | 1.2650 [0.7416;2.1578] | 0.3883 |
| Jonmarker et al, 2020 | 1.3644 [0.8058;2.3103] | 0.2475 |
| Helms et al, 2021 | 1.3891 [0.8188;2.3565] | 0.2230 |
| Kodama et al, 2021 | 1.1304 [0.7153;1.7864] | 0.5995 |
| Longhitano et al, 2020 | 1.3694 [0.8080;2.3210] | 0.2428 |
| Lopes et al, 2021 | 1.3563 [0.7899;2.3288] | 0.2692 |
| Marco et al, 2021 | 1.1591 [0.7089;1.8953] | 0.5561 |
| Martinelli et al, 2021 | 1.3330 [0.7785;2.2826] | 0.2949 |
| Lawler et al, 2021 | 1.3880 [0.8217;2.3444] | 0.2203 |
| Perepu et al, 2021 | 1.3010 [0.7675;2.2052] | 0.3284 |
| Pesavento et al, 2020 | 1.2986 [0.7731;2.1813] | 0.3234 |
| Voicu et al, 2021 | 1.3221 [0.7756;2.2536] | 0.3048 |
| Takayama et al, 2021 | 1.3816 [0.8313;2.2963] | 0.2123 |
| Vaughn et al, 2021 | 1.1328 [0.7381;1.7384] | 0.5684 |
