# Supplementary material for: Intermediate-to-therapeutic versus prophylactic anticoagulation for coagulopathy in hospitalized COVID-19 patients: a systemic review and meta-analysis
Source: Thromb J. 2021 Nov 24;19:91. doi: 10.1186/s12959-021-00343-1 (PMC8611638; doi:10.1186/s12959-021-00343-1)

**Additional file 14. Funnel plot for the assessment risk of publication bias of thrombotic complication events outcome**
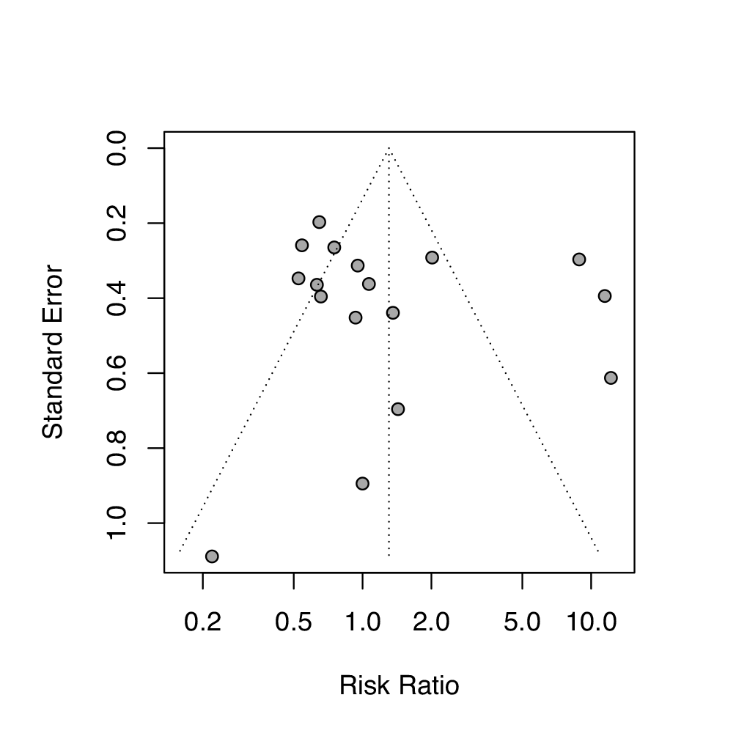

Supplement: Supplementary file 14 — Additional file 14. Funnel plot for the assessment risk of publication bias of thrombotic complication events outcome. [file 12959_2021_343_MOESM14_ESM.docx]
