# Supplementary material for: Intermediate-to-therapeutic versus prophylactic anticoagulation for coagulopathy in hospitalized COVID-19 patients: a systemic review and meta-analysis
Source: Thromb J. 2021 Nov 24;19:91. doi: 10.1186/s12959-021-00343-1 (PMC8611638; doi:10.1186/s12959-021-00343-1)
Supplement: Supplementary file 15 — Additional file 15. The quality of evidence for thrombotic complication events assessed by GRADE framework. [file 12959_2021_343_MOESM15_ESM.docx]

**Additional file 15. The quality of evidence for thrombotic complication events assessed by GRADE framework**

| **Certainty assessment** | | | | | | | **NO. of patients** | | **Effect** | | **Certainty** | **Importance** |
| --- | --- | --- | --- | --- | --- | --- | --- | --- | --- | --- | --- | --- |
| **NO. of studies** | **Study design** | **Risk of bias** | **Inconsistency** | **Indirectness** | **Imprecision** | **Other considerations** | **intermediate-to-therapeutic dose AC** | **prophylactic dose AC** | **Relative (95% CI)** | **Absolute (95% CI)** |  |  |
| **Thrombotic events** | | | | | | | | | | | | |
| 17 | observational studies and RCTs | serious | serious | not serious | not serious | none | 244/3397 (7.2%) | 306/5095 (6.0%) | **RR 1.30** (0.79 to 2.15) | **18 more per 1,000** (from 13 fewer to 69 more) | ⨁◯◯◯ VERY LOW | CRITICAL |
| **Thrombotic events in critically ill patients admitted to ICU** | | | | | | | | | | | | |
| 7 | observational studies and RCTs | not serious | not serious | not serious | not serious | all plausible residual confounding would reduce the demonstrated effect | 100/1165 (8.6%) | 162/1250 (13.0%) | **RR 0.71** (0.56 to 0.89) | **38 fewer per 1,000** (from 57 fewer to 14 fewer) | ⨁⨁⨁◯ MODERATE | CRITICAL |
